# Deep learning segmentation of the choroid plexus from structural magnetic resonance imaging (MRI): validation and normative ranges across the adult lifespan

**DOI:** 10.21203/rs.3.rs-3338860/v1

**Published:** 2023-09-13

**Authors:** Jarrod J. Eisma, Colin D. McKnight, Kilian Hett, Jason Elenberger, Alexander K. Song, Ciaran Considine, Daniel O. Claassen, Manus J. Donahue

**Affiliations:** Vanderbilt University Medical Center; Vanderbilt University Medical Center; Vanderbilt University Medical Center; Vanderbilt University Medical Center; Vanderbilt University Medical Center; Vanderbilt University Medical Center; Vanderbilt University Medical Center; Vanderbilt University Medical Center

**Keywords:** choroid plexus, deep learning, glymphatic, segmentation, cerebrospinal fluid, neurofluids

## Abstract

**Background:**

The choroid plexus functions as the blood-cerebrospinal fluid barrier, plays an important role in neurofluid production and circulation, and has gained increased attention in light of the recent elucidation of neurofluid circulation dysfunction in neurodegenerative conditions. However, methods for routinely quantifying choroid plexus volume are suboptimal and require technical improvements and validation. Here, we propose three deep learning models that can segment the choroid plexus from commonly-acquired anatomical MRI data and report performance metrics and changes across the adult lifespan.

**Methods:**

Fully convolutional neural networks were trained from 3-D T_1_-weighted, 3-D T_2_-weighted, and 2-D T_2_-weighted FLAIR MRI and gold-standard manual segmentations in healthy and neurodegenerative participants across the lifespan (n=50; age=21–85 years). Dice coefficients, 95% Hausdorff distances, and area-under-curve (AUCs) were calculated for each model and compared to segmentations from FreeSurfer using two-tailed Wilcoxon tests (significance criteria: p<0.05 after false discovery rate multiple comparisons correction). Metrics were regressed against lateral ventricular volume using generalized linear models to assess model performance for varying levels of atrophy. Finally, models were applied to an expanded cohort of healthy adults (n=98; age=21–89 years) to provide an exemplar of choroid plexus volumetry values across the lifespan.

**Results:**

Deep learning results yielded Dice coefficient=0.72, Hausdorff distance=1.97 mm, AUC=0.87 for T_1_-weighted MRI, Dice coefficient=0.72, Hausdorff distance=2.22 mm, AUC=0.87 for T_2_-weighted MRI, and Dice coefficient=0.74, Hausdorff distance=1.69 mm, AUC=0.87 for T_2_-weighted FLAIR MRI; values did not differ significantly between2 MRI sequences and were statistically improved compared to current commercially-available algorithms (p<0.001). The intraclass coefficients were 0.95, 0.95, and 0.96 between T_1_-weighted and T_2_-FLAIR, T_1_-weighted and T_2_-weighted, and T_2_-weighted and T_2_-FLAIR models, respectively. Mean lateral ventricle choroid plexus volume across all participants was 3.20±1.4 cm^3^; a significant, positive relationship (R^2^=0.54; slope=0.047) was observed between participant age and choroid plexus volume for all MRI sequences (p<0.001).

**Conclusions:**

Findings support comparable performance in choroid plexus delineation between standard, clinically available, non-contrasted anatomical MRI sequences. The software embedding the evaluated models is freely available online and should provide a useful tool for the growing number of studies that desire to quantitatively evaluate choroid plexus structure and function (https://github.com/hettk/chp_seg).

## Background

The choroid plexus consists of a collection of fenestrated capillaries located in all four ventricles, with the majority of the choroid plexus volume residing in the atria of the lateral ventricles. The choroid plexus is widely believed to be the primary source of cerebrospinal fluid (CSF) production in the brain, producing CSF at a rate of 430–530 mL/day^1^ and has gained additional recent attention owing to its role as one of the most proximal components of the brain’s waste clearance system with potential relevance to brain neurotoxic metabolite and protein accumulation^2^.

The choroid plexus has been well characterized from animal studies, but how choroid plexus structure and function change in the context of disease^3–5^ and aging^6–7^ in humans is an area of active and emerging interest. In the context of aging, it has been shown that choroid plexus volume increases, and perfusion decreases, with advanced age^7–8^. Diffusion-weighted magnetic resonance imaging (MRI) has also revealed that choroid plexus mean diffusivity increases, and fractional anisotropy decreases, with advanced age^8^. Increasing choroid plexus volume may relate to increasing severity of cognitive impairment in the spectrum of Alzheimer’s disease related disorders^3^ and in support of this possibility, reduced choroid plexus metabolism from ^18^Fluoro-deoxyglucose positron emission tomography (PET) has been reported in patients with Alzheimer’s disease compared to patients with amnestic mild cognitive impairment and healthy controls^10^. Perfusion-weighted arterial spin labelling MRI has been utilized further to characterize choroid plexus response to various pharmacological stimuli^9^, which may aid in evaluating novel therapeutics for brain disorders that may implicate the choroid plexus such as dementia.

However, one limitation to the advancement of neuroimaging studies of the choroid plexus is the lack of an accurate, automatic tool to segment the structure from anatomical images. Manual segmentations, as with other tissues, are impractical in large cohort studies and the choroid plexus has varying appearances on standard MRI sequences due to its heterogeneous relaxometry characteristics, making manual segmentations an even more difficult process. Many neuroimaging software packages do not include segmentation options for the choroid plexus, and those that do include choroid plexus segmentation tools have been reported to have suboptimal performance in many applications^11^. Fully convolutional neural networks (FCNN) have shown state-of-the-art performance for segmentation of other brain structures^12^, and recent work has proposed deep learning-based methods to segment the choroid plexus^11,13–14^. These methods rely on 3-D magnetization-prepared-rapid-gradient-echo (MPRAGE) T1-weighted MRI to learn choroid plexus anatomical patterns, however, this approach may provide sub-optimal contrast for choroid plexus visualization and quantification given limited contrast between hypointense CSF signal and normo-to-mildly hypointense choroid plexus signal. In addition to T_1_-weighted MRI, T_2_-weighted and T_2_-weighted FLuid Attenuated Inversion Recovery (FLAIR) MRI also are commonly acquired in both clinical and research neuroimaging environments and may have differing segmentation accuracy.

In this study, we aim to develop and evaluate automated tools for segmenting the choroid plexus from three types of commonly-acquired MRI sequences (i.e. T_1_-weighted, T_2_-weighted, and T_2_-weighted FLAIR) and compare the results from these methods to gold-standard manual tracings and to the commonly used neuroimaging analysis software, FreeSurfer. We also evaluate performance of these methods in an additional cohort of healthy controls to report how the choroid plexus evolves across the adult lifespan, which will provide an exemplar for future clinical studies which may implicate the choroid plexus, such as Alzheimer’s disease, Parkinson’s disease, multiple sclerosis, and traumatic brain injury. The code is made publicly available for academic use.

## Methods

### Demographics

This study had two components. First, we developed and evaluated a deep learning algorithm using separate standard MRI sequences in a diverse cohort of adults (including ages and conditions, as detailed below) with the intent of providing a generalizable segmentation algorithm. Second, we applied the method to healthy adults across the lifespan to provide an exemplar for choroid plexus volumes.

Adult participants (n = 50 for model training; n = 98 for subsequent healthy control lifespan analysis) provided informed, written consent in accordance with the Vanderbilt University Institutional Review Board (IRB) and the Declaration of Helsinki and its amendments. All participants were enrolled between February 2020 and July 2023. It is well-known that the brain atrophies with advancing age and in various neurological disorders, and with this atrophy comes ventricular enlargement and often associated choroid plexus enlargement^7–8^. In order to make the proposed method as generalizable as possible, for algorithm training and development we deliberately enrolled a heterogeneous cohort of persons across the adult lifespan. Participants included healthy controls and patients with mild cognitive impairment (MCI), Alzheimer’s disease, Parkinson’s disease, and Huntington’s disease. Inclusion criteria for healthy control participants consisted of no history of cerebrovascular disease, anemia, psychosis, or neurological disorder including but not limited to prior overt stroke, sickle cell anemia, schizophrenia, bipolar disorder, Alzheimer’s disease, Parkinson’s disease, or multiple sclerosis. The presence of non-specific white matter lesions was not an exclusion criterion for healthy controls, as these lesions become more prevalent with aging, and we sought our cohort to be generalizable and representative. Diagnosis of Alzheimer’s disease, mild cognitive impairment, Parkinson’s disease, and Huntington’s disease was made by a board-certified neurologist (DOC; experience = 15 years).

### Image acquisition

All participants underwent non-contrasted MRI at 3 Tesla with body coil radiofrequency transmission and 32-channel SENSE-array reception on a Philips Ingenia system (Philips Healthcare, Best, The Netherlands). Anatomical images consisted of: (i) 3-D T_1_-weighted MPRAGE (TR = 8.1 ms; TE = 3.7 ms; field of view = 256 × 180 × 150 mm^3^; number of slices = 150; spatial resolution = 1.0 × 1.0 × 1.0 mm^3^; duration = 4 minutes 32 seconds), (ii) 2-D T_2_-weighted fluid-attenuated-inversion-recovery (FLAIR) turbo-spin-echo (TR = 11,000 ms; TE = 120 ms; TI = 2800 ms; field of view = 230 × 184 × 144 mm^3^; number of slices = 29; spatial resolution = 0.57 × 0.57 × 4.0 mm^3^; duration = 1 minute 39 seconds), and (iii) 3-D T_2_-weighted turbo-spin-echo (TR = 2500 ms; TE = 331 ms; field of view = 250 × 250 × 189 mm^3^; number of slices = 242; spatial resolution = 0.78 × 0.78 × 0.78 mm^3^; duration = 4 minutes 8 seconds).

### Manual segmentation of the choroid plexus

Data utilized for manual segmentation of the choroid plexus consisted of 3-D T_1_-weighted, 2-D axial T_2_-FLAIR-weighted, and 3-D T_2_-weighted MRI from 50 enrolled subjects. Ground truth choroid plexus segmentation was performed manually with final approval from a board-certified neuroradiologist (CDM; experience = 9 years). The manual delineation protocol was defined as follows: first, 2-D axial T_2_-FLAIR and 3-D T_2_-weighted images were co-registered to 3-D T_1_-weighted images using linear registration tools from the Advanced Normalization Tools (ANTs) software package^15^. Finally, multi-modal data were utilized by the rater to delineate the choroid plexus using FMRIB Software Library (FSL)^16^. Manual segmentations focused on the choroid plexus in the atria of the lateral ventricles. To limit biasing of the deep learning method, delineations were careful to not include partial volumes from nearby anatomical structures including the thalamus and periventricular white matter.

### Automatic choroid plexus segmentation

Automatic choroid plexus segmentations were generated via a fully convolutional neural network model.

The machine learning model was designed following a U-NET architecture^17^. This architecture was chosen because of its proven success in medical image segmentation algorithms and consisted of an encoding and a decoding step. The encoding step was composed of three blocks each composed of two layers. The number of filters was set to 64 for the first block and doubled at each block thereafter. Each layer consised of a 3-D convolution (kernel size = 3×3×3 voxels, stride = 1, and padding = 1), a batch normalization, followed by a rectified linear unit. Feature maps from each block were downsampled using a maximum pooling operation (kernel size = 2×2×2 voxels). The decoding step followed the same architecture, with each block dividing the number of filters by 2. Up-sampling between each decoding block was performed with a 3-D transposed convolution (kernel size = 2×2×2 voxels, stride = 2×2×2 voxels, and no padding). The final segmentation map was obtained using a block composed of a 3-D convolution operator (kernel size = 1×1×1 voxels, stride = 1×1×1 voxels, and no padding) followed by a hyperbolic tangent as activation function. The model was trained on three separate datasets to compare performance across different MRI sequences (i.e., T_1_-weighted, T_2_-weighted, and T_2_-FLAIR-weighted images). All images were registered non-linearly with ANTs software to the International Consortium for Brain Mapping-Montreal Neurological Institute (ICBM-MNI) 152 T_1_-weigthed template^18^. Non-linear registration was utilized in this step-in order to reduce morphological variability of the lateral ventricles and thus increase the inter-subject similarity of the choroid plexus appearance.

### Implementation details

**Figure 1:**
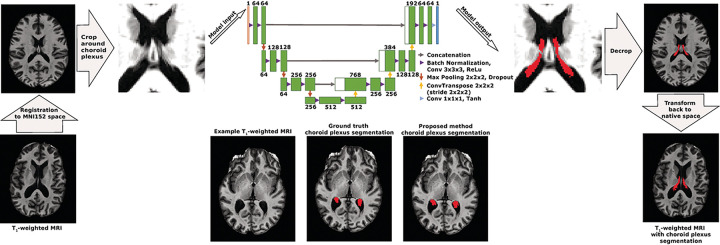
Overview of the processing pipeline of the anatomical magnetic resonance imaging (MRI) utilized in the proposed deep learning method. Examples are shown for a T_1_-weigthed MRI, but this pipeline also was utilized for T_2_-weighted and T_2_-weighted fluid-attenuated inversion recovery (FLAIR) MRI. Input images were registered to MNI152 space and cropped around the choroid plexus based off a probabilistic atlas generated from ground truth manual choroid plexus segmentations. Cropped images were then used as training input for the 3-D U-NET fully convolutional neural network. The number of inputs for each trained model was 1 and the number of output structures was 1 (i.e., choroid plexus). Cropped outputs were then decropped and inverse transformed to the native imaging space. Example images are shown from a 69-year-old male with Parkinson’s disease. (MRI: magnetic resonance imaging; Conv: convolution; ReLu: rectified linear unit; Tanh: hyperbolic tangent)

A patch-based approach for training of the machine learning model was employed. Patches of 64×64×64 voxels were extracted from the MNI-registered images, and these patches were centered on random voxels from the choroid plexus probabilistic atlas that was generated from the average of the manual choroid plexus segmentations included in the training data set. In total, 41 overlapping patches from each participant were used to train the model. During the training phase, random flipping along the longitudinal fissure was implemented to increase the training sample size further. The network was trained using an ADAM^19^ optimizer with a learning rate set to 10^−4^. A generalized Dice loss function was used to train the network^20^. Lastly, the segmentation mask patches were pieced back together in MNI space and transformed back to the native *T*_1_-weighted space using the inverse transformation and nearest-neighbor interpolation. An overview of the processing pipeline with a diagram of the 3-D U-NET architecture is shown in Fig. 1.

For comparison to available algorithms, choroid plexus segmentation masks were generated using FreeSurfer’s standard segmentation procedure from T_1_-weighted MRI in all training subjects^21–22^. Briefly, input images were skull-stripped and intensity corrected, and then FreeSurfer’s *aseg* atlas was used to generate left and right choroid plexus labels. These labels were inverse transformed back to each subject’s native T_1_ space and combined to form one choroid plexus mask per subject. These masks were then compared to ground truth manual segmentations for statistical analysis.

Lastly, for the lifespan volumetry analysis, T_1_, T_2_, and T_2_-FLAIR images were separately preprocessed as described previously, and the deep learning model for each corresponding MRI sequence was utilized to generate choroid plexus segmentations for all enrolled participants. From these segmentations in each participant’s native imaging space, the choroid plexus volume was calculated in cm^3^.

### Statistical analyses

To evaluate the accuracy of the choroid plexus segmentation, we investigated how the model performed when trained with different sets of MRI sequences (i.e., T_1_-weighted, T_2_-weighted, and T_2_-FLAIR images). For each MRI sequence, a 5-fold cross-validation scheme was implemented with 30 participants utilized in model training, 10 participants utilized in model validation, and 10 participants utilized in model evaluation. Pseudo-randomization was used to ensure the same participant groups across each modality-based model.

To verify the accuracy of the machine learning and FreeSurfer outputs, standard comparison metrics between the ground truth segmentation and machine learning output were calculated. The Dice-Sørensen coefficient, 95% Hausdorff distance, and area under curve (AUC) were calculated for each iteration of cross-validation and averaged across the iterations to produce representative metrics for each modality-based model. These metrics were then compared to FreeSurfer using two-tailed Wilcoxon tests.

As an exploratory analysis, we evaluated these performance metrics as a function of participant lateral ventricular volume to gain more understanding on how the machine learning models perform across in different anatomical environments. Lateral ventricular volume was calculated from each participant’s T_1_-weighted MRI using the AssemblyNet software package^23^. Generalized linear models were utilized to separately regress performance metrics against model-testing participants’ lateral ventricular volume. The intraclass correlation coefficient between the choroid plexus volume for each healthy control participant from the three deep learning models and between each of the choroid plexus volumes for the training participants and their ground truth choroid plexus volumes, were calculated and descriptive statistics presented as Bland-Altman plots.

For the lifespan component of this study, a generalized linear model was utilized to regress the choroid plexus volume from each modality model against participants’ age. Sex was included as a covariate as well in this regression to account for previously found sex-dependence on choroid plexus volume^4^, and total intracranial volume calculated from AssemblyNet was included as a covariate as well. The McFadden R^2^ values were calculated for each regression model.

The machine learning algorithm was implemented using the PyTorch Python library^24^, and pre-processing, post-processing, and statistical analyses were implemented in Matlab^25^. All statistical analyses were implemented using the R software package^26^. All p-values were corrected with false discovery rate for multiple comparisons correction^27^. Significance criteria was defined as *p* < 0.05.

## Results

### Demographics: algorithm development

Participants (n = 50) included in the training, validation, and testing of the machine learning models ranged in age from 21 to 85 years, included 27 males and 23 females, and 29 healthy participants and 21 participants with neurodegeneration (Supplementary Table 1).

### Algorithm performance metrics

Performance metrics for each proposed machine learning model, and a previously available FreeSurfer algorithm, are reported in Table 1. The average Dice coefficients were 0.72, 0.72, and 0.74 for the T_1_-weighted, T_2_-weighted, and T_2_-weighted-FLAIR models, respectively, while the average Dice coefficient for the FreeSurfer output applied to the T_1_-weighted image was 0.19. Two-tailed Wilcoxon tests revealed a significant difference in the Dice coefficient between the T_1_-weighted machine learning method and FreeSurfer, the T_2_-weighted machine learning method and FreeSurfer, and the T_2_-weighted-FLAIR machine learning method and FreeSurfer (all p-values < 0.001).

The average 95% Hausdorff distances were 1.97, 2.22, and 1.69 mm for the T_1_-weighted, T_2_-weighted, and T_2_-weighted-FLAIR models, respectively, and the average 95% Hausdorff distance for the FreeSurfer output was 10.4 mm. Two-tailed Wilcoxon tests revealed a significant difference in the 95% Hausdorff distance between the T_1_-weighted machine learning method and FreeSurfer, the T_2_-weighted machine learning method and FreeSurfer, and the T_2_-weighted-FLAIR machine learning method and FreeSurfer (all p-values < 0.001).

The average AUCs were 0.87 for each of the models and the average AUC for the FreeSurfer output was 0.56. Two-tailed Wilcoxon tests revealed a significant difference in the AUC between the T_1_-weighted machine learning method and FreeSurfer, the T_2_-weighted machine learning method and FreeSurfer, and the T_2_-weighted-FLAIR machine learning method and FreeSurfer (all p-values < 0.001).

**Figure 2:**
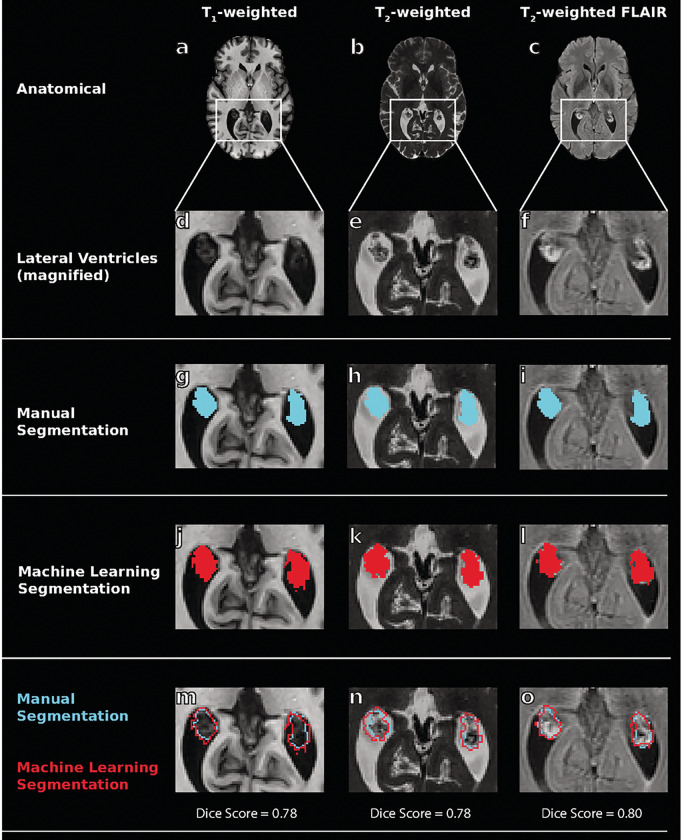
Example choroid plexus segmentations from machine learning models in a 53-year-old male with mild cognitive impairment. From left to right, columns show results from T_1_-weighted images, T_2_-weighted images, and T_2_-FLAIR-weighted images. The first row (panels a-c) shows the anatomical magnetic resonance images (MRI) utilized in this study for deep learning training, and the second row (d-f) shows these same images zoomed on the lateral ventricles where the choroid plexus resides. The remaining rows show the manual segmentations (g-i), machine learning output segmentations (j-l), and the overlay (m-o) of these segmentations for each type of MRI. The dice scores of each model (T_1_: 0.78, T_2_: 0.78, T_2_-FLAIR: 0.80) are shown and reflect consistently accurate performance across MRI sequences. (FLAIR: FLuid-Attenuated Inversion Recovery)

**Figure 3:**
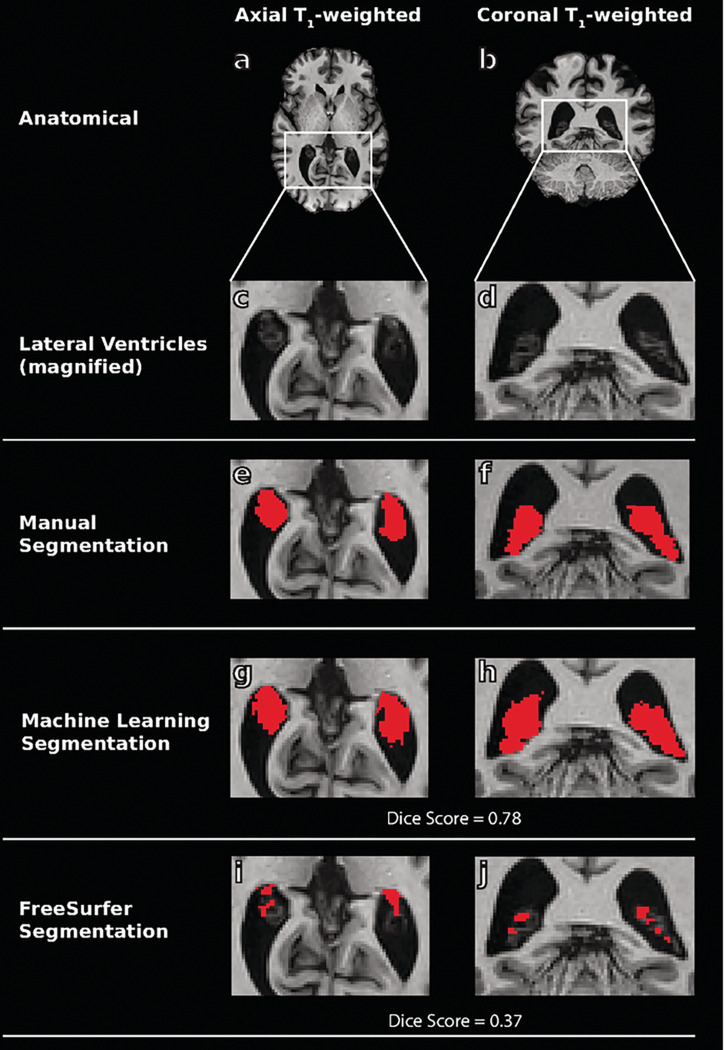
Example T_1_-weighted magnetic resonance images (MRI) from a 53-year-old male with mild cognitive impairment (a-d) and manual tracings (e-f) utilized in training of the machine learning methods. Example outputs from the T_1_-weighted-trained machine learning model (g-h) are shown compared to FreeSurfer segmentations (i-j). Dice scores are shown for machine learning and FreeSurfer outputs and reflect an improvement in segmentation accuracy for the proposed method.

An example of each machine learning model output compared to ground truth and FreeSurfer choroid plexus segmentations from a 53-year-old male with MCI are shown in Figs. 2 and 3.

**Figure 4:**
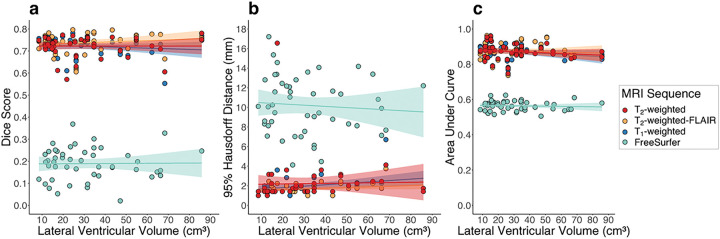
Regression plots for machine learning dice score (a), 95% Hausdorff distance (b), and AUC (c) against testing subject lateral ventricular volume. Overall, models performed consistently across lateral ventricular volume. The only model metric that was significantly related to lateral ventricular volume was the T_1_-weighted model’s 95% Hausdorff distance (ß value= 0.015; p-value=0.05). (MRI: magnetic resonance imaging; FLAIR: fluid-attenuated inversion recovery).

We also investigated the relationship between model performance and lateral ventricular volume. Numerical results and graphical representations of these relationships are shown in Supplementary Table 2 and Fig. 4, respectively. For each MRI sequence, the lateral ventricular volume of the testing participant was not significantly related to the Dice coefficient (T_1_-weighted p-value: 0.44; T_2_-weighted p-value: 0.99; T_2_-weighted-FLAIR p-value: 0.40). For the T_2_-weighted and T_2_-weighted-FLAIR models, the lateral ventricular volume was not significantly related to the 95% Hausdorff Distance (T_2_-weighted p-value: 0.92; T_2_-weighted-FLAIR p-value: 0.094); however, for the T_1_-weighted model, the lateral ventricular volume was positively related to the 95% Hausdorff Distance (p-value: 0.050). For each MRI sequence, the lateral ventricular volume was not significantly related to the AUC (T_1_-weighted p-value: 0.20; T_2_-weighted p-value: 0.35; T_2_-weighted-FLAIR p-value: 0.69). For the FreeSurfer outputs, none of the metrics related to ventricular volume (Dice p-value: 0.99; 95% Hausdorff distance p-value: 0.44; AUC p-value: 0.69).

**Figure 5:**
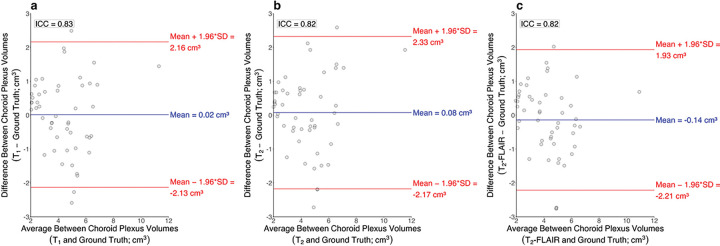
Bland Altman plots for choroid plexus volumes generated from T_1_-weighted deep learning methods (a), T_2_-weighted deep learning methods (b), and T_2_-weighted-FLAIR deep learning methods (c) compared to the ground truth manual segmentation volumes. The intraclass correlation coefficient between T_1_-weighted and ground truth choroid plexus volumes was 0.83, T_2_-weighted and ground truth choroid plexus volumes was 0.82, and T_2_-weighted-FLAIR and ground truth choroid plexus volumes was 0.82.

Intraclass correlation coefficients were 0.83, 0.82, and 0.82 between T_1_-weighted deep learning choroid plexus volumes and ground truth choroid plexus volumes (Fig. 5a), T_2_-weighted deep learning choroid plexus volumes and ground truth choroid plexus volumes (Fig. 5b), and T_2_-weighted-FLAIR deep learning choroid plexus volumes and ground truth choroid plexus volumes (Fig. 5c), respectively.

### Choroid plexus volume and age

Participants (n = 98) included in the assessment of choroid plexus volume across the adult lifespan ranged from 21 to 89 years of age and included 46 males and 52 females (Supplementary Table 3).

**Figure 6:**
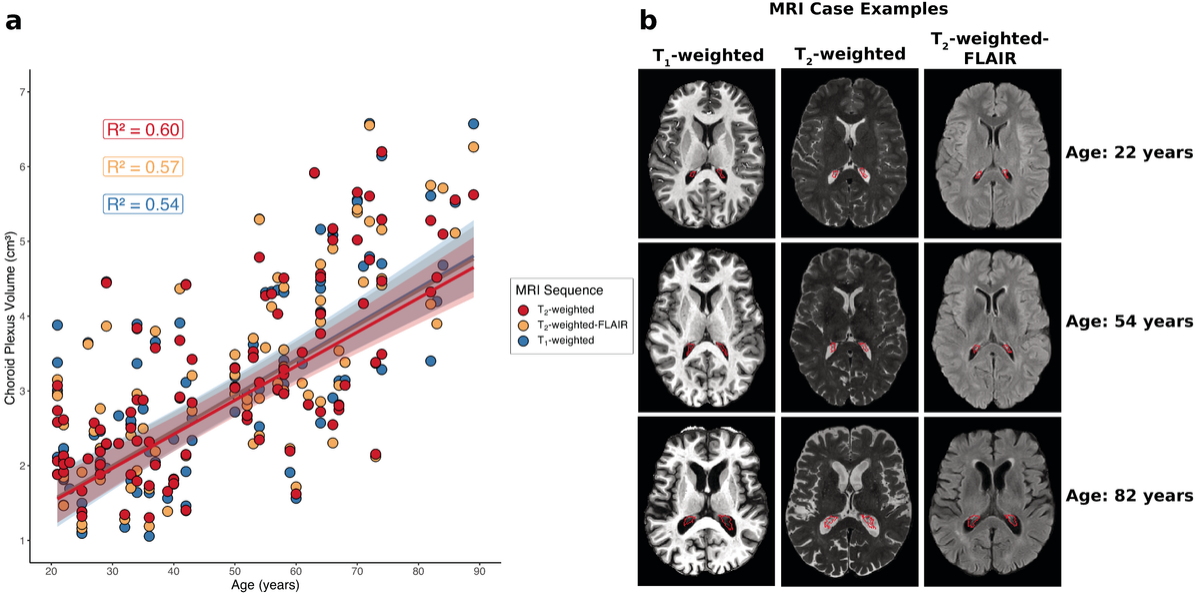
(a) Regression plot displaying choroid plexus volume against subjects’ age for each MRI modality in healthy controls. McFadden’s R^2^ values are reported for each regression model. (b) Case examples for younger, middle, and older-aged healthy controls showing an increase in choroid plexus volume with age. Results show consistently across MRI types that choroid plexus volume increases with age across the adult lifespan. (FLAIR: FLuid-Attenuated Inversion Recovery).

**Figure 7:**
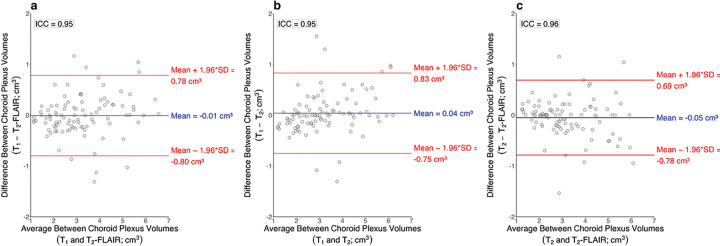
Bland Altman plots for choroid plexus volumes generated from T_1_-weighted and T_2_-weighted-FLAIR deep learning methods (a), T_1_-weighted and T_2_-weighted deep learning methods (b), and T_2_-weighted and T_2_-weighted-FLAIR deep learning methods (c). The intraclass correlation coefficient between T_1_-weighted and T_2_-weighted-FLAIR choroid plexus volumes was 0.95, T_1_-weighted and T_2_-weighted choroid plexus volumes was 0.95, and T_2_-weighted and T_2_-weighted-FLAIR choroid plexus volumes was 0.96.

Numerical and graphical results from these regression analyses are shown in Supplementary Table 4 and Fig. 6, respectively. For each MRI sequence, participant age was positively related to choroid plexus volume (all p-values < 0.001). Additionally, for each MRI sequence, participant sex was significantly related to choroid plexus volume, with males having a larger choroid plexus volume than females (T_1_-weighted p-value: 0.0012; T_2_-weighted and T_2_-weighted-FLAIR p-values < 0.001). For each MRI sequence, total intracranial volume was not significantly related to choroid plexus volume (T_1_-weighted p-value: 0.094; T_2_-weighted p-value: 0.094; T_2_-weighted-FLAIR p-value: 0.11). The McFadden’s R^2^ values for the T_1_-weighted, T_2_-weighted, and T_2_-weighted-FLAIR regression models were 0.54, 0.60, and 0.57, respectively. Intraclass correlation coefficients between choroid plexus volumes were 0.95, 0.95, and 0.96 for T_1_-weighted and T_2_-weighted-FLAIR deep learning methods (Fig. 7a), T_1_-weighted and T_2_-weighted deep learning methods (Fig. 7b), and T_2_-weighted and T_2_-weighted-FLAIR deep learning methods (Fig. 7c). Representative choroid plexus volumes across the adult lifespan are included in Supplementary Table 3.

## Discussion

A deep learning method with 3-D U-NET architecture was trained for automatic segmentation of the choroid plexus from standard anatomical MRI. Models were trained separately on three types of commonly-acquired images: T_1_-weighted, T_2_-weighted, and T_2_-weighted-FLAIR MRI from a cohort of 50 participants across the adult lifespan and with differing levels of tissue atrophy. The findings of the study support improved segmentation of the choroid plexus using the proposed method compared to currently-available software, and also provides an exemplar of choroid plexus volumes, as a function of age, that may provide a reference for studies in neurodegeneration. The software is also made freely available for academic use.

The three proposed deep learning methods were able to segment the choroid plexus with Dice coefficients, 95% Hausdorff distances, and AUC values comparable to those found in literature^11^. We expand on the method provided by Zhao and colleagues, which utilized T_1_-weighted MRI to train a 3-D U-NET model with improved performance compared to FreeSurfer^11^, by including additional anatomical MRI that is commonly acquired in clinical settings, specifically 3-D T_2_-weighted and 2-D T_2_-weighted-FLAIR MRI. The proposed methods also showed improved performance compared to automatic segmentations from FreeSurfer across all calculated metrics, an important finding as many previous and ongoing studies utilize FreeSurfer for choroid plexus volumetric analyses^3,28–29^. FreeSurfer utilizes an atlas-based segmentation approach, whereby a manually labeled training set provided by the software is used to estimate probabilistic neuroanatomical labels for each voxel in the MRI volume registered to this atlas^21^. While this software has shown robust sensitivity for segmentation of many noncortical structures^21–22^, the results from this study and from Zhao et al. suggest that it may not be the most accurate for choroid plexus segmentation, possibly due to the inter-subject variation in choroid plexus structure^11^. Further, we found that most of the proposed models perform accurately independent of lateral ventricular volume. All model performance metrics had no significant relationship to testing subject lateral ventricular volume except the T_1_ model’s 95% Hausdorff distance. Observing the central plot in Fig. 4, it is possible that this relationship was driven by a statistical outlier. The observation that the other performance metrics were relatively stable in the presence of a variety of lateral ventricular volumes provides further support for the robustness of these models.

Additionally, we applied these deep learning methods in a cohort of 98 healthy controls across the adult lifespan and found a significant positive relationship between subject age and choroid plexus volume with all three methods. Intraclass correlation coefficients between these volumes were high, suggesting consistently accurate calculations of choroid plexus volumes between models in this cohort. Intraclass coefficients between training subjects’ ground truth choroid plexus volumes and deep learning choroid plexus volumes were also high, suggesting accurate segmentation performance from the proposed methods. We also reported normative ranges of choroid plexus volume across the adult lifespan and found an approximate 15% increase in choroid plexus volume with each decade of life on average across all MRI sequences, which agrees with previous reports from literature^7,28^. Previous reported age-related increases in choroid plexus volume using similar methods as described in this study and age-related decreases in choroid plexus perfusion detected from arterial spin labeling MRI^7^. Sun and colleagues recently reported age-related increases in choroid plexus volume using manual delineations from T_1_-weighted MRI and enlarged stromal tissue in the choroid plexus of older subjects using ultrasmall superparamagnetic iron oxide (USPIO)-enhanced high resolution 2D gradient echo MRI at 7 Tesla^30^. Previous histopathological studies using hematoxylin-eosin staining have shown a thickened vascular wall and fibrotic stroma in the choroid plexus of elderly subjects as well^31^, which could explain the enlarged volume on anatomical MRI.

While these methods showed robust results and provided findings that aligned with previous reports from literature, several factors should be considered when interpreting the results. The training data sample size included 50 participants. However, the chroid plexus was segmented using gold-standard manual segmentation by a radiologist and we chose a 3-D U-NET architecture which has shown robust accuracy with limited data set samples^11,17^. We also adopted a data augmentation strategy and utilized a patch-based approach and random flipping, which increased the training dataset from 50 to 4100 samples. Furthernore, we included participants in the training dataset with and without clinically diagnosed neurodegenerative diseases to increase generalizability. Additionally, the lifespan study reports on trends in choroid plexus volume with age, and participants are approximately equally distributed across the adult lifespan. However, this study was cross-sectional and not longitudinal (e.g., following the same participant over time) and also may be underpowered to infer small changes in choroid plexus volume over limited age epochs (e.g., a decade of life or less). Future work could expand on this cohort, using large data sets, to address these issues more rigorously.

## Conclusion

We propose a deep learning segmentation method for automatic segmentation of the choroid plexus from the following standard anatomical MRI: T_1_-weighted, T_2_-weighted, and T_2_-weighted-FLAIR. The proposed method performs similarly across these three commonly-acquired MRI sequences and improves segmentation accuracy compared to commercially available algorithms. Finally, we provide ranges for healthy lateral ventricle choroid plexus volume across the lifespan, which should provide a useful exemplar for future work that aims to identify pathological aberrations in choroid plexus volume and function. The proposed method is also made freely available for academic use.

## Figures and Tables

**Table 1:** Performance metrics for each machine learning method and FreeSurfer using manual segmentations as the ground truth. Values are shown as mean (range). Metrics for the machine learning-based methods were calculated from 10 pseudo-random testing participants across 5 cross-validation iterations, whereas metrics for FreeSurfer were calculated from all 50 participants included in the algorithm development.

Method	Sørensen-Dice Coefficient	95% Hausdorff Distance (mm)	AUC
Deep Learning from T -weighted MRI	0.72 (*0.55–0.78*)***	1.97 (*1.00–6.71*)***	0.87 (*0.75–0.96*)***
Deep Learning from T -weighted MRI	0.72 (*0.57–0.78*)***	2.22 (*1.00–16.6*)***	0.87 (*0.75–0.96*)***
Deep Learning from T -weighted- FLAIR MRI	0.74 (*0.61–0.80*)***	1.69 (*1.00–3.74*)***	0.87 (*0.74–0.96*)***
FreeSurfer from T -weighted MRI	0.19 (*0.02–0.37*)	10.4 (*4.12–17.2*)	0.56 (*0.50–0.62*)

*** indicates two-tailed Wilcoxon test revealed a significant difference between the machine learning method and FreeSurfer (p-value< 0.001).

## Data Availability

The data that support the findings of this study are available from the corresponding author, MJD, upon reasonable request. Choroid plexus segmentation software is freely available for public use at: https://github.com/hettk/chp_seg

## Abbreviations

ANTs: Advanced Normalization Tools
AUC: area under curve
CSF: cerebrospinal fluid
FCNN: fully convolutional neural network
FLAIR: fluid attenuated inversion recovery
FSL: FMRIB Software Library
ICBM-MNI: International Consortium for Brain Mapping-Montreal Neurological Institute
IRB: Institutional Review Board
MCI: mild cognitive impairmen
MPRAGE: magnetization-prepared-rapid-gradient-echo
MRI: magnetic resonance imaging/images
PET: positron emission tomography
TE: echo time
TR: repetition time
USPIO: ultrasmall superparamagnetic iron oxide
